# High-Dose Dexamethasone Manipulates the Tumor Microenvironment and Internal Metabolic Pathways in Anti-Tumor Progression

**DOI:** 10.3390/ijms21051846

**Published:** 2020-03-07

**Authors:** Lei Xu, Hua Xia, Dongsheng Ni, Yanxia Hu, Jianing Liu, Yao Qin, Qin Zhou, Qiying Yi, Yajun Xie

**Affiliations:** 1 The Ministry of Education Key Laboratory of Clinical Diagnostics, School of Laboratory Medicine, Chongqing Medical University, Chongqing 400016, China; xulei@stu.cqmu.edu.cn (L.X.); xiahua@stu.cqmu.edu.cn (H.X.); dongshengni@stu.cqmu.edu.cn (D.N.); yanxiahu@stu.cqmu.edu.cn (Y.H.); keithliu@stu.cqmu.edu.cn (J.L.); qinyao@stu.cqmu.edu.cn (Y.Q.); zhouqin@cqmu.edu.cn (Q.Z.); 2 Laboratory Animal Center, Chongqing Medical University, Chongqing 400016, China

**Keywords:** dexamethasone, FAO, FFA, glycolysis metabolism, Pparα, Pparg, TG synthesis, tumor progression

## Abstract

High-dose dexamethasone (DEX) is used to treat chemotherapy-induced nausea and vomiting or to control immunotherapy-related autoimmune diseases in clinical practice. However, the underlying mechanisms of high-dose DEX in tumor progression remain unaddressed. Therefore, we explored the effects of high-dose DEX on tumor progression and the potential mechanisms of its anti-tumor function using immunohistochemistry, histological examination, real-time quantitative PCR (qPCR), and Western blotting. Tumor volume, blood vessel invasion, and levels of the cell proliferation markers Ki67 and c-Myc and the anti-apoptotic marker Bcl2 decreased in response to high-dose DEX. However, the cell apoptosis marker cleaved caspase 3 increased significantly in mice treated with 50 mg/kg DEX compared with controls. Some genes associated with immune responses were significantly downregulated following treatment with 50 mg/kg DEX e.g., *Cxcl9*, *Cxcl10*, *Cd3e*, *Gzmb*, *Ifng*, *Foxp3*, *S100a9*, *Arg1*, and *Mrc1*. In contrast, the M1-like tumor-associated macrophages (TAMs) activation marker *Nos2* was shown to be increased. Moreover, the expression of peroxisome proliferator-activated receptors α and γ (*Pparα* and *Pparg*, respectively) was shown to be significantly upregulated in livers or tumors treated with DEX. However, high-dose DEX treatment decreased the expression of glucose and lipid metabolic pathway-related genes such as glycolysis-associated genes (*Glut1*, *Hk2*, *Pgk1*, *Idh3a*), triglyceride (TG) synthesis genes (*Gpam*, *Agpat2*, *Dgat1*), exogenous free fatty acid (FFA) uptake-related genes (*Fabp1*, *Slc27a4*, and *CD36*), and fatty acid oxidation (FAO) genes (*Acadm*, *Acaa1*, *Cpt1a*, *Pnpla2*). In addition, increased serum glucose and decreased serum TG and non-esterified fatty acid (NEFA) were observed in DEX treated-xenografted tumor mice. These findings indicate that high-dose DEX-inhibited tumor progression is a complicated process, not only activated by M1-like TAMs, but also decreased by the uptake and consumption of glucose and lipids that block the raw material and energy supply of cancer cells. Activated M1-like TAMs and inefficient glucose and lipid metabolism delayed tumor cell growth and promoted apoptosis. These findings have important implications for the application of DEX combined with drugs that target key metabolism pathways for tumor therapy in clinical practice.

## 1. Introduction

Synthetic glucocorticoids (GCs) such as dexamethasone (DEX) have been widely used as chemotherapy adjuvant drugs to treat related symptoms including nausea, vomiting, and fatigue in patients with solid tumors [1,2]. However, the role of DEX in the treatment of solid tumors remains controversial. Previous studies showed that DEX suppresses prostate cancer growth by decreasing vascular endothelial growth factor (VEGF) and IL-8 production and inhibiting tumor angiogenesis [3]. Low-dose DEX also inhibits ovarian tumor growth by upregulating microRNA-708 expression [4]. Evidence also demonstrates that DEX promotes cancer progression by regulating cell proliferation-associated signaling pathways e.g., p53 signal pathway inactivation and p38-MAPK pathway activation [5]. These data demonstrate the role of low-dose DEX (5–100 μg/kg body weight) in tumor growth in vivo. Compared with low-dose DEX, high-dose DEX is widely used in clinical practice owing to its effectiveness in protecting patients from vomiting, especially in patients with advanced solid tumors following chemotherapy [6,7].

As a promising anti-tumor treatment, immunotherapy enhances the innate immune response by regulation of immune checkpoints [8]. However, it is associated with a number of immune-associated adverse effects such as autoimmune disorders [9]. Adjuvant treatment with high-dose DEX is an effective way to combat the side effects of immunotherapy [10]. However, the immunosuppressive role of DEX seems to contrast with its role in anti-tumor progression. These conflicting observations led us to explore the role of DEX in anti-tumor progression aside from its role as an immunosuppressant. Recent evidence demonstrates that DEX inhibits immune cell function by affecting CD8^+^ T cells, regulatory T cells (Tregs), and myeloid-derived suppressor cells (MDSCs) [11,12]. In addition, long-term and high-dose dexamethasone treatment decreases the expression of IGHV genes in the mouse spleen [13]. A switch from anti-tumor M1-like tumor-associated macrophages (TAMs) to pro-tumor M2-like TAMs in the tumor microenvironment (TME) is helpful for tumor progression, but no evidence has shown that DEX is associated with M1- to M2-like TAMs transformation. In addition to its role as an immunosuppressant, DEX also affects the regulation of glucose and fatty acid metabolism. Previous studies suggest that DEX inhibits glucose uptake or decreases phosphoenolpyruvate carboxykinase 2 (PCK2) expression to promote tumor cell death or apoptosis [12,14]. Moreover, DEX downregulates the expression of liver fatty acid binding-associated genes and inhibits the expression of the liver mitochondrial matrix acyl-CoA dehydrogenases in mice, which are relevant to free fatty acid (FFA) import and fatty acid oxidation (FAO), respectively [15,16]. Glucose uptake and transport provide the main raw material for cells to synthesize amino acids, nucleotides, lipids, and adenosine triphosphate (ATP) which are necessary for survival and reproduction of cancer cells [17,18,19]. Malignant cells depend on rapid de novo fatty acid synthesis to support their development and growth [20]. Apart from endogenous lipid biosynthesis, cancer cells show exacerbated exogenous FFA uptake for ATP production via FAO [21,22]. Therefore, blocking the energy or material supply of cancer cells is an effective treatment strategy. DEX acts as an immunosuppressant and metabolic regulation reagent, and high doses of DEX are widely used in clinical practice for cancer therapy. However, the concrete role of high-dose DEX in anti-tumor progression remains unknown.

Here, we constructed a xenografted tumor model by hypodermic injection of Lewis lung carcinoma (LLC) cells into C57BL/6 mice and demonstrated that high-dose DEX treatment significantly inhibited tumor progression and increased survival time in tumor-bearing mice. High-dose DEX activated M1-like TAMs and decreased glucose and lipid uptake and consumption in tumors leading to the inhibition of cell growth and promotion of apoptosis.

## 2. Results

### 2.1. High-Dose DEX Injection Inhibited Tumor Progression and Increased Survival Time in Mice with Xenografted Tumors

**Figure 1:**
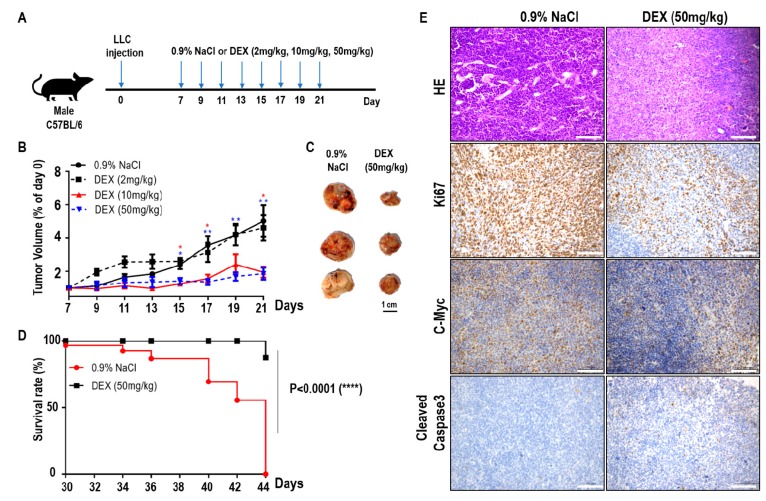
High-dose dexamethasone (DEX) injection inhibited tumor progression and increased survival. (**A**) Schematic of the DEX treatment schedule. Mice were inoculated subcutaneously with 5 × 10^5^ Lewis lung carcinoma (LLC) cells, and on days 7, 9, 11, 13, 15, 17, 19, and 21 post-inoculation, mice were intraperitoneally treated with 0.9% NaCl or various doses of DEX (2 mg/kg, 10 mg/kg, and 50 mg/kg). (**B**) Representative tumor growth in mice treated with 0.9% NaCl or various doses of DEX (*n* = 5–6 mice per group). Data are presented as mean ± SEM. Statistical significance was analyzed by unpaired Student’s *t* test and indicated by *, *p* < 0.05; **, *p* < 0.01. (**C**) Representative tumor size in mice treated with 0.9% NaCl or 50 mg/kg DEX. (**D**) Representative survival curves of the percentages of mice in treatment with 0.9% NaCl or 50 mg/kg DEX (*n* = 8 mice per group). (**E**) Tumors were excised from mice treated with 0.9% NaCl or 50 mg/kg DEX on day 21 of the schedule presented in (**A**), followed by hematoxylin and eosin staining of blood vessel invasion and immunohistochemistry staining of Ki67, c-Myc, and cleaved caspase-3. All scale bars represent 100 μm.

To explore the effects of DEX on tumor progression, we constructed a xenografted tumor model by hypodermic injection of LLC cells into C57BL/6 mice. Seven days later, the mice with established tumors were randomized and injected intraperitoneally with various doses of DEX or 0.9% NaCl every two days (Figure 1A). As the DEX concentration increased (2 mg/kg, 10 mg/kg, and 50 mg/kg body weight), the tumor volume markedly decreased and tumor progression was significantly delayed, especially for the DEX (50 mg/kg)-injected group (Figure 1B,C). In addition, the survival time of tumor-bearing mice increased significantly in the 50 mg/kg group compared with controls (Figure 1D). Hematoxylin and eosin staining of tumors from 50 mg/kg DEX-injected mice also suggested a significant decrease in blood vessel invasion compared with mice injected with 0.9% NaCl (Figure 1E). Immunohistochemical analysis showed that high-dose DEX injection led to decreased expression of the cell proliferation markers Ki67 and c-Myc, and increased the expression of cell apoptosis marker cleaved caspase 3 (Figure 1E). Our results indicate that high-dose DEX injection decreased tumor aggression as demonstrated by decreased tumor angiogenesis and proliferation and increased apoptosis.

### 2.2. High-Dose DEX Injection Inhibited Most Immune Cell Responses but Promoted M1-Like TAMs Polarization and Proliferator-Activated Receptor γ (Pparg) Expression in the TME

**Figure 2:**
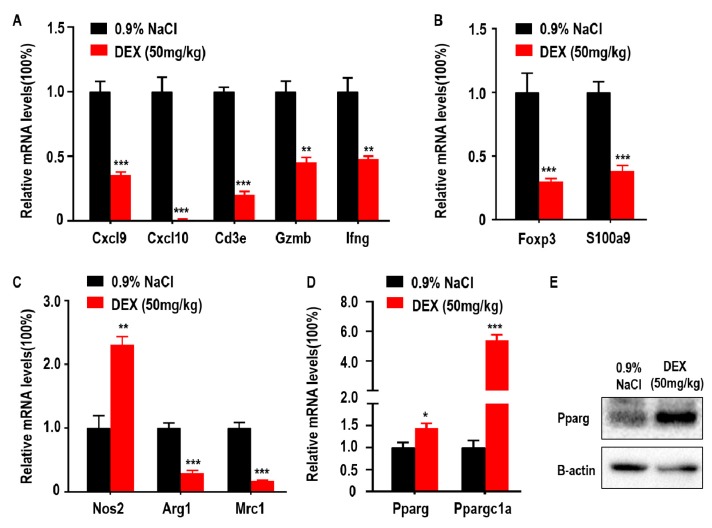
High-dose DEX inhibited anti- or pro-tumor immune response in most cells, but promoted M1-like tumor-associated macrophages (TAMs) polarization and peroxisome proliferator-activated receptor γ (Pparg) expression in the tumor microenvironment (TME). (**A**) Intratumoral genes relevant to the anti-tumor T cell-mediated antitumor immune response. (**B**) Protumor immune cell regulatory T cells (Tregs) and myeloid-derived suppressor cells (MDSCs) markers. (**C**) Antitumor M1-like TAMs or protumor M2-like TAMs markers. (**D**,**E**) The expressions of Pparg and Ppargc1a were determined via qPCR or Western blotting of tumors taken from C57BL/6 mice treated with 0.9% NaCl or 50 mg/kg DEX. All data are presented as mean ± SEM and *p* < 0.05 is considered significant (*p < 0.05, **p < 0.01, ***p < 0.001; *n* = 3 mice per group). Results representative of three independent experiments.

DEX has been previously shown to downregulate genes coding antibody heavy chain region and has also been identified as an immune response inhibitor in clinical therapy [13]. In addition, adaptive immune control of tumor growth relies on the accumulation and function of anti-tumor immune CD8^+^ T cells in the tumor microenvironment (TME) [11]. Some suppressor cells, such as MDSCs and Tregs, are also recruited to the TME by tumor cells and inhibit anti-tumor immune responses and promote tumor progression [23]. Furthermore, tumor progression is associated with a functional switch from anti-tumor M1-like TAMs to pro-tumor M2-like TAMs in the TME. To explore the association between DEX inhibited-tumor growth and accumulation of immune response-related cells, we measured the levels of *Cxcl9*, *Cxcl10*, *Cd3e*, *Gzmb*, and *Ifng* which are closely related to the recruitment, presence, and function of Th1 cells and CD8^+^ T cells in the TME. We found that the expression of *Cxcl9*, *Cxcl10*, *Cd3e*, *Gzmb*, and *Ifng* were decreased following DEX treatment (Figure 2A). Foxp3 is a transcriptional regulator, which is pivotal for the development and function of Tregs, and S100a9 is a proinflammatory molecule that is positively associated with the accumulation and immunosuppressive function of MDSCs in the TME [24]. Both genes were significantly reduced after DEX treatment (Figure 2B). Mrc1 and Arg1 expression indicate the polarization of M2 macrophages, and Nos2 is used as a marker of M1 macrophages polarization [25,26]. Mrc1 and Arg1 were decreased; however, Nos2 expression increased in tumors from the DEX-treated group (Figure 2C). We also measured glucose and lipid metabolism by qPCR as demonstrated by changes in Pparg and Ppargc1a expression. Here we found that Pparg expression was significantly increased, which was confirmed by immunoblotting assay (Figure 2D,E). Furthermore, Ppargc1a, which enhances Pparg transcriptional activity, was also upregulated in tumors from the DEX treatment group (Figure 2D).

### 2.3. High-Dose DEX Inhibited Tumor Progression Accompanied with Glucose and Lipid Metabolism Suppression in the TME

**Figure 3:**
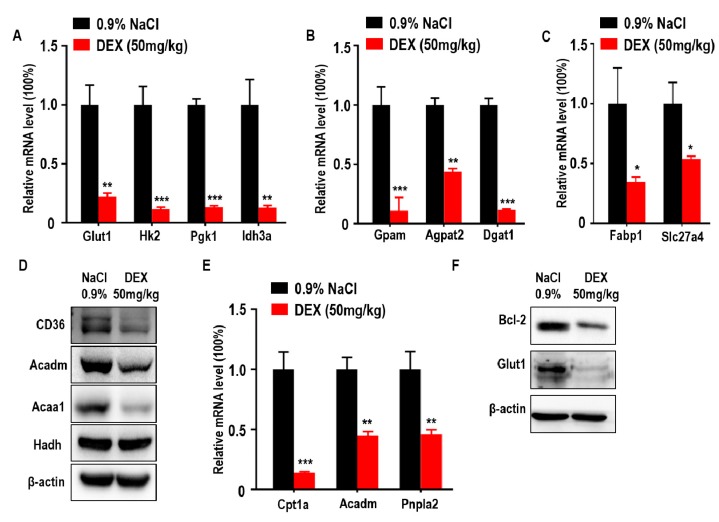
High-dose DEX injection inhibited the expression of enzymes relevant to glycolysis, triglyceride (TG) synthesis, free fatty acid (FFA) import, and fatty acid oxidation (FAO). (**A**) Quantitative RT-PCR analysis of genes relevant to glucose transporter and glycolysis enzymes in tumors from mice after treatment with 0.9% NaCl or DEX. (**B**) Quantitative RT-PCR analysis of glycerol-3-phosphate acyltransferase (*Gpam*), 1-acylglycerol-3-phosphate O-acyltransferase (*Agpat2*), and diacylglycerol O-acyltransferase 1 (*Dgat1*) in tumors from mice after treatment with 0.9% NaCl or DEX. (**C**) Quantitative RT-PCR analysis of fatty acid binding protein 1 (*Fabp1*) and solute carrier family 27 member 4 (*Slc27a4*) in tumors from mice after treatment with 0.9% NaCl or DEX. (**D**) Immunoblot analysis of CD36 and FAO pathway enzymes in tumors treated with 0.9% NaCl or DEX (50 mg/kg), respectively. (**E**) Quantitative RT-PCR analysis of genes relevant to FAO pathway enzymes in tumors from mice after treatment with 0.9% NaCl or DEX. (**F**) Immunoblot analysis of Bcl-2 and glucose transporter 1 (Glut1) in tumors treatment with 0.9% NaCl or DEX (50 mg/kg), respectively. Results representative of three independent experiments. All data are presented as mean ± SEM and *p* < 0.05 is considered significant (*p < 0.05, **p < 0.01, ***p < 0.001; *n* = 3 mice per group).

Glucose is an important nutritional component of blood and a metabolic substrate for organism survival and growth [27]. Deficiency of glucose uptake or consumption disrupts metabolic balance and promotes cell death [28,29]. To further explore the association between DEX-inhibited tumor growth with glucose metabolism, we measured the expression of glucose metabolism-associated genes. We found that glucose transporter 1 (*Glut1*) and glycolytic and tricarboxylic acid cycle (TCA) markers hexokinase 2 (*Hk2*), phosphoglycerate kinase 1 (*Pgk1*), and isocitrate dehydrogenase subunit alpha (*Idh3a*) were downregulated in response to DEX (Figure 3A). Lipids are also important for cancer cell metabolism and provide energy to drive tumor growth; therefore, we also analyzed key genes involved in lipid metabolism and found that TG synthesis-related genes were decreased in DEX-treated groups, including glycerol-3-phosphate acyltransferase (*Gpam*), 1-acylglycerol-3-phosphate O-acyltransferase 2 (*Agpat2*), and diacylglycerol O-acyltransferase 1 (*Dgat1*) (Figure 3B). In addition to TG synthesis, exogenous FFA uptake and consumption is necessary for energy production [30]. We also found FFA import-associated genes were decreased, such as fatty acid binding protein 1 (*Fabp1*) and solute carrier family 27 member 4 (*Slc27a4*), which was further confirmed by immunoblotting analysis of CD36 (Figure 3C,D). Moreover, tumor cells take up these fatty acids to produce ATP via FAO [21]. In DEX-treated groups, the expression of enzymes involved in FAO were significantly reduced, including carnitine palmitoyltransferase 1A (*Cpt1a*), acyl-CoA dehydrogenase medium chain (*Acadm*), patatin-like phospholipase domain containing 2 (*Pnpla2*), and acetyl-CoA acyltransferase 1 (Acaa1); however, the level of hydroxyacyl-CoA dehydrogenase (Hadh) remained unchanged compared to control (Figure 3D,E). The ability to evade apoptosis is a key characteristic of cancer cells [31]. A decrease in glucose uptake and consumption can induce the expression of pro-apoptotic factors and promote intrinsic apoptosis [28,29]. Further, Pparg has also been shown to have proapoptotic effects [32]. We showed that the expression of Bcl2, a classical regulator of anti-apoptosis in tumors, was significantly decreased in response to DEX treatment (Figure 3F).

### 2.4. DEX Treatment Decreased the Expression of Enzymes Relevant to Glucose and Lipid Metabolism in Cultured Cells

**Figure 4:**
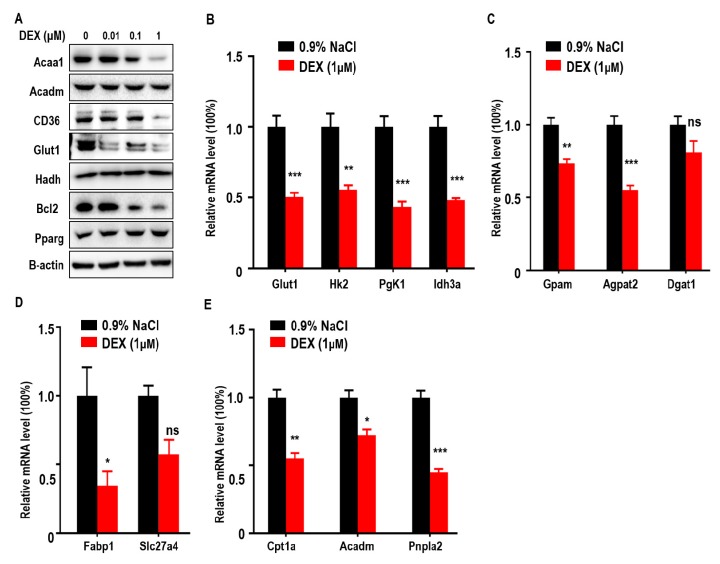
Treatment with DEX inhibits metabolism enzyme levels relevant to glycolysis, TG synthesis, FFA import, and FAO, and upregulates Pparg levels in vitro. (**A**) Immunoblot analysis of Acaa1, Acadm, CD36, Glut1, hydroxyacyl-CoA dehydrogenase (Hadh), Pparg, and Bcl2 in LLC cells treated with 0.9% NaCl or various doses of DEX (0.01, 0.1, 1 μM), respectively. (**B**–**E**) Quantitative RT-PCR analysis of genes relevant to glycolysis pathway enzymes (**B**), TG synthesis (**C**), FFA import (**D**), and the FAO pathway (**E**), in LLC cells after treatment with 0.9% NaCl or 1 μM DEX, respectively. All data are presented as mean ± SEM and *p* < 0.05 is considered significant (*p < 0.05, **p < 0.01, ***p < 0.001).

To further confirm the effects of DEX on glucose and lipid metabolism in LLC cells, we measured expressions of Acaa1, Acadm, CD36, Glut1, and Bcl-2, which were all reduced after DEX treatment whereas Pparg expression increased proportionally with dose (Figure 4A). qPCR assays also showed that DEX (1 μM) treatment downregulated glycolysis (*Hk2*, *Pgk1*, *Idh3a*), TG synthesis (*Gpam*, *Agpat2*), FFA import (*Fabp1*), and FAO pathway-related genes (*Cpt1a*, *Acadm*, *Pnpla2*) (Figure 4B–4E). Consistent with in vivo experiment, these results demonstrate that DEX increases Pparg levels and inhibits the expression of enzymes relevant to TG synthesis, exogenous FFA import, and the FAO pathway.

### 2.5. High-Dose DEX Treatment Significantly Reduced Serum Triglyceride and Non-Esterified Fatty Acid (NEFA) Levels and Increased Serum Glucose Levels in Tumor-Bearing Mice

**Figure 5:**
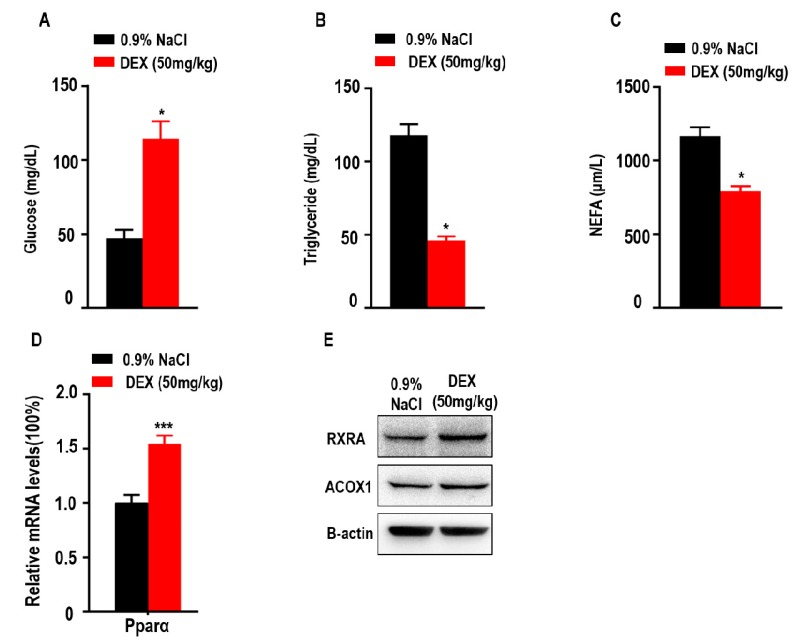
Treatment with high-dose DEX significantly reduced serum triglyceride and non-esterified fatty acid (NEFA) levels, and increased serum glucose levels in tumor-bearing mice. Quantification of serum glucose (**A**), triglycerides (**B**), and NEFAs (**C**) in tumor-bearing mice (*n* = 3 mice per group) after treatment with 0.9% NaCl and 50 mg/kg DEX. (**D**,**E**) The expressions of proliferator-activated receptor α (Pparα), retinoid X receptor alpha (RXRA), and peroxisomal acyl-coenzyme A oxidase 1 (ACOX1) were determined via qPCR or Western blotting of livers taken from C57BL/6 mice treated with 0.9% NaCl or 50 mg/kg DEX. All data are presented as mean ± SEM and *p* < 0.05 is considered significant (*p < 0.05, **p < 0.01, ***p < 0.001; *n* = 3 mice per group). Results are representative of three independent experiments.

Since glucose, triglycerides, and NEFAs play an important role in metabolism as energy sources, we explored the levels of glucose, TGs and NEFAs in tumor-bearing mice after DEX treatment. Here, we found treatment with high-dose DEX significantly increased the levels of serum glucose in tumor-bearing mice (Figure 5A). We also found treatment with high-dose DEX significantly decreased the levels of serum TGs and NEFAs in tumor-bearing mice (Figure 5B,C). Proliferator-activated receptor-alpha (Pparα) stimulated fatty acid beta oxidation and promoted the metabolism of NEFAs and TGs [33,34]. The retinoid X receptor alpha (RXRA)/ Pparα heterodimer is required for Pparα transcriptional activity on fatty acid oxidation genes such as peroxisomal acyl-coenzyme A oxidase 1 (ACOX1). We also found that the expression of Pparα, RXRA, and ACOX1 was increased in liver (Figure 5D,E).

## 3. Discussion

In clinical practice, DEX has been widely used as an adjuvant to chemotherapy or immunotherapy to treat some adverse reactions such as vomiting and autoimmune diseases, especially for patients with advanced solid tumors [1,10]; however, the dosage of DEX in clinical therapy varies greatly and the efficacy and side effects associated with each dosage remain unknown. Here, we assessed the efficacy and safety of a sufficient dose of DEX in tumor progression and explored the role of high-dose DEX in solid tumor progression. We found that high-dose DEX treatment significantly inhibited tumor progression and promoted survival in mice, especially those treated with 50 mg/kg of DEX; however, the same dosage sigificantly inhibited the accumulation of most immune cells in the TME, except enhancing the transformation of M2- to M1-like TAMs. Moreover, the uptake and consumption of glucose and lipid was downregulated in DEX-treated tumor or cultured cells and the classic anti-apoptotic marker Bcl2 was significantly inhibited in tumors. This study evaluated the association between high-dose DEX and tumor progression via its effects on immune cells and internal metabolic pathways.

DEX has been shown to inhibit tumor cell growth in vitro and vivo [3,12]. However, in prior studies the authors constructed an immunocompromised transplantable mouse model which did not have the mutual effects of immune system and metobolism pathways in tumor progression. Here, we demonstrated that high-dose DEX inhibits tumor progression in the immunocompetent mouse model. Evidence suggests that DEX leads to decreased levels of VEGF and inhibits intratumoral angiogenesis during anti-tumor progression [3]. In line with this, we show here that high-dose DEX alleviated blood vessel invasion. DEX inhibits cancer cell proliferation by downregulation of c-Myc and promotes apoptosis in the treatment of malignancies [12,35]. We found that treatment with high-dose DEX remarkably decreased expression of the proliferation markers ki67 and c-Myc and the anti-apoptotic marker Bcl2. We also showed that high-dose DEX increased expression of the pro-apoptosis marker cleaved caspase 3. These results collectively show that high dose DEX decreases tumor malignancy.

DEX has been shown to inhibit immune system function and we demonstrated that high-dose DEX significantly inhibits the expression of genes encoding antibody heavychain region and acts as an immune response inhibitor in clinical therapy [13,36]. Immune cells play an important role in maintaining tumor progression in the TME including the regulation of antitumor immune cells (CD8^+^T, M1-like TAMs) and protumor immune cells (Tregs, MDSCs, and M2-like TAMs) [37]. We also found that high-dose DEX treatment significantly decreased the expression of intratumoral anti-tumor immune T cells function markers *Cxcl9*, *Cxcl10*, *Gzmb*, *Ifng*, and *Cd3e*. Tregs and MDSCs are immunosuppressive cells that are associated with anti-tumor immunosuppression [38]. TAMs are polarized in tumor with features more closely resembling the pro-tumorigenic M2 phenotype rather than the anti-tumor M1 phenotype [39]. Previous studies reported that Arg1 and Mrc1 are markers of M2 macrophage polarization and Nos2 is the marker of M1 macrophage polarization [40,41]. The effects of TAMs on tumor cells depend on two phenotypes: tumor-promoting M2-like TAMs and the tumoricidal M1-like TAMs [42]. Previous studies showed that low-dose DEX significantly reduced the number of TAM and MDSCs in the TME [4]; we further demonstrated that high-dose DEX significantly decreased the levels of the intratumoral immunosuppressive cell markers *Foxp3* and *S100a9*. The levels of *Arg1* and *Mrc1* were significantly decreased and *Nos2* levels were significantly increased following high-dose DEX treatment. These observations demonstratred the dual function of high-dose DEX on immune cells. In the past decades, DEX has been recognized as immunosuppressive reagent in clinical practice [11,12], but our findings prove that DEX has different functions (inhibition or promotion) in different immune cell subtypes. This is a good explanation for the seemingly contradictory results observed in tumors treated with DEX.

Cancer cells depend on the uptake and consumption of glucose and lipids, and DEX has been shown to regulate glucose and fatty acid metabolism [14,15]. Our qPCR and immunoblotting analysis confirmed that high-dose DEX increased the expression of Pparg in tumors. Moreover, important proteins involved in aerobic glycolysis such as Glut1, Hk2, Pgk1, and Idh3a were decreased in tumors. Previous studies demonstrated that activation of Pparg could impair the aerobic glycoysis process via inhibiting Pgk1 and Glut1 function [18,43]. These findings indicated that high-dose DEX-inhibited tumor growth via the reduction of glucose metabolism enzymes expression, which starves the cancer cells of ATP and important amino acids/nucleic acid precursors. Gpam, Agpat2, and Dgat1 are critical enzymes involved in TG biosynthesis and Gpam overexpression correlates with cancer cell migration [44]. DEX injection decreases the hepatic TG accumulation in goats [45]. Here, we found that high-dose DEX treatment decreased the expression of TG synthesis-related enzymes (*Gpam*, *Agpat2*, *Dgat1*). Fatty acid transporter proteins (CD36, *Fabp1*, *Slc27a4*) mediate the uptake of fatty acids and were reported to be highly expressed in cancer cells and to function in tumor growth [46]. Previous studies reported downregulation of L-Arabinose binding protein (LABP) expression in rats treated with DEX [16]. We found that high-dose DEX also decreased the expression of FFA import-associated genes *CD36*, *Fabp1*, and *Slc27a4*. DEX treatment also decreased fatty acid β-oxidation pathway-associated genes including *Cpt1a*, *Acadm*, *Pnpla2*, and *Acaa1*. Previous studies demonstrated that DEX decreased the expression of liver Cpt1 in vivo [47], and inhibits the expression of Acadm and liver lipid secretion in mice [15]. DEX was also shown to decrease the levels of Pnpla2, a lipolysis gene expressed by preadipocytes [48]. Moreover, high-dose DEX significantly increased serum glucose levels and decreased the levels of serum TG and NEFA in tumor-bearing mice. In clinical practice, DEX is generally thought to increase the expression of TG and NEFA. Since the liver is an important organ for TG synthesis, we found that Pparα was significantly increased after high-dose DEX treatment in liver. Pparα stimulated fatty acid beta oxidation and promoted the metabolism of NEFA and TG [33,34]. The RXRA/ Pparα heterodimer is required for Pparα transcriptional activity on fatty acid oxidation genes such as ACOX1. We also found the expression of RXRA and ACOX1 was increased in liver after high-dose DEX treatment. Integrating previous reports and our findings, we found that high-dose DEX disrupts the balance of glucose and lipid metabolism in tumors. However, metabolism provides raw material and energey for tumors and deficiency of glucose and lipid metabolism promotes apoptosis through increasing pro-apoptotic genes or decreasing anti-apoptotic genes such as Bcl2 [14,49,50]. This was confirmed in our study where high-dose DEX treatment promoted Pparg and inhibited Bcl2 expression.

Based on these results, we conclude that high-dose DEX-inhibited tumor progression is a complicated process that not only activates M1-like TAMs but also disrupts the uptake and consumption of glucose and lipids, which blocks the raw material and energy supply of cancer cells. Finally, activated M1-like TAMs and inefficient glucose and lipid metabolism delayed tumor cell growth and promoted tumor cell apoptosis. These findings are benificial to the application of high-dose DEX as an adjuvant treatment to chemotherapy or immunotherapy in patients with advanced tumors in clinical practice.

## 4. Materials and Methods

### 4.1. Ethics Statement

All animal experiments were approved by the Institutional Animal Care and Use Committee of Chongqing Medical University (Reference Number: 2018020, Date Approved: 6 June 2018). All mice were maintained in a special pathogen-free facility. All efforts were made to minimize animal suffering.

### 4.2. Sources of Mice and Cells

C57BL/6 mice (6–8 weeks old) were obtained from the laboratory animal center of Chongqing Medical University (Chongqing, China). Mice were raised in specific pathogen-free facilities and allowed free access to food and water. The murine Lewis lung carcinoma (LLC) cell line was a gift obtained by Xiaoping Chen professor from the Guangzhou Institutes of Biomedicine and Health, Chinese Academy of Sciences. Cells were cultured in RPMI 1640 (Gibco, Carlsbad, CA, USA) supplemented with 10% fetal bovine serum (Gibco, Carlsbad, CA, USA) and 1% penicillin/streptomycin (Invitrogen, Grand Island, NY, USA) in a humidified atmosphere of 5% CO_2_ at 37 °C.

### 4.3. Tumor Models and In Vivo Treatments

5 × 10^5^ LLC cells were injected subcutaneously into the right flank of mice. When tumors became visible at 7 days, C57BL/6 mice were randomized into four groups of 5–6 mice each. There were no significant differences in tumor size between the groups. Tumor-bearing mice were intraperitoneally injected with various dose DEX (Cisen, Jining, Shandong, China) (2 mg/kg, 10 mg/kg, and 50 mg/kg body weight) or 0.9% NaCl (vehicle) every other day for a total of eight injections from day 7. Tumor size was measured every two days with an electronic caliper and calculated as (length) × (width) × (width)/2. Tumors were harvested 21 days post-inoculation for further analysis. Survival was measured using the Kaplan–Meier method (GraphPad Software, lnc., La Jolla, CA, USA) in mice treated with 0.9% NaCl and 50 mg/kg DEX, respectively (*n* = 8 mice per group).

### 4.4. Treatment with DEX In Vitro

The LLC cells grown to the log phase were inoculated into 6 well plates at 2 × 10^5^ per well and placed in the cell incubator. After incubating for 24 h, the cells were randomly divided into four groups, and the medium was replaced with fresh complete medium containing 0.9% NaCl or DEX (0.01, 0.1, 1 μM), and then placed in the cell incubator for 24 h. All cells were harvested for further experiments.

### 4.5. RNA Isolation and qPCR

Total RNA was extracted from tissues (tumor or liver) and cells using TRIzol Reagent (Invitrogen, Carlsbad, CA, USA). For this, 2 μg of RNA were reverse transcribed into cDNA by the First Strand cDNA Synthesis Kit (Thermo Scientific, Waltham, MA, USA). The mRNA level was detected by UltraSYBR Mixture (CWBIO, Guangzhou, China) and normalized relative to the 18s mRNA levels. Primer sequences (5’-3’) used were: Cxcl9 (accession number: NM_008599.4): GGA GTT CGA GGA ACC CTA GTG and GGG ATT TGT AGT GGA TCG TGC; Cxcl10 (accession number: NM_021274.2): CCA AGT GCT GCC GTC ATT TTC and GGC TCG CAG GGA TGA TTT CAA; Cd3e (accession number: NM_007648.5): GGT GCT CCA GGA TTT CTC GG and GCC TTG GCC TTC CTA TTC TTG; Gzmb (accession number: NM_013542.3): TCA TGC TGC TAA AGC TGA AGA G and CCC GCA CAT ATC TGA TTG GTT T; Ifng (accession number: NM_008337.4): ATG AAC GCT ACA CAC TGC ATC and CCA TCC TTT TGC CAG TTC CTC; Foxp3 (accession number: NM_054039): CAC CTA TGC CAC CCT TAT CCG and CAT GCG AGT AAA CCA ATG GTA GA; S100a9 (accession number: NM_001281852.1): ATA CTC TAG GAA GGA AGG ACA CC and TCC ATG ATG TCA TTT ATG AGG GC; Nos2 (accession number: NM_010927): GGA GTG ACG GCA AAC ATG ACT and TCG ATG CAC AAC TGG GTG AAC; Arg1 (accession number: NM_007482.3): CTC CAA GCC AAA GTC CTT AGA G and AGG AGC TGT CAT TAG GGA CAT C; Mrc1 (accession number: NM_008559): CTA TGC GCT GCG TTA TCA CAG and AAA GAA AGT GAC GAG GCA GAG; Pparg (accession number: NM_001127330.2): TCG CTG ATG CAC TGC CTA TG and GAG AGG TCC ACA GAG CTG ATT; Ppargc1a (accession number: NM_008904.2): TAT GGA GTG ACA TAG AGT GTG CT and CCA CTT CAA TCC ACC CAG AAA G; Glut1 (accession number: NM_011400.3): TGT GGG AGG AGC AGT GCT CG and TGG GCT CTC CGT AGC GGT G; Hk2 (accession number: NM_013820.3): TGA TCG CCT GCT TAT TCA CGG and ACC GCC TAG AAA TCT CCA GAA GG; Pgk1 (accession number: NM_008828.3): ATG TCG CTT TCC AAC AAG CTG and TGG CTC CAT TGT CCA AGC AG; Idh3a (accession number: NM_029573.2): TGG GTG TCC AAG GTC TCT CG and TCT GGG CCA ATT CCA TCT CC; Gpam (accession number: NM_001356285.1): ACA GTT GGC ACA ATA GAC GTT T and CCT TCC ATT TCA GTG TTG CAG A; Agpat2 (accession number: NM_026212): CAG CCA GGT TCT ACG CCA AG and TGA TGC TCA TGT TAT CCA CGG T; Dgat1 (accession number: NM_010046.3): TCC GTC CAG GGT GGT AGT G and TGA ACA AAG AAT CTT GCA GAC GA; Fabp1 (accession number: NM_017399.5): ATG AAC TTC TCC GGC AAG TAC C and CTG ACA CCC CCT TGA TGT CC; Slc27a4 (accession number: NM_011989.5): TGA GTT TGT GGG TCT GTG GCT AGG and AAG ACA GTG GCG CAG GGC ATC; Cpt1a (accession number: NM_013495.2): CTC CGC CTG AGC CAT GAA G and CAC CAG TGA TGA TGC CAT TCT; Acadm (accession number: NM_007382.5): ATG CCT GTG ATT CTT GCT GGA and ACA TCT TCT GGC CGT TGA TAA C; Pnpla2 (accession number: NM_025802.3): TTC CCG AGG GAG ACC AAG TG and TGC CGA GGC TCC GTA GAT G; Pparα (accession number: NM_011144.6): AGC CCC ATC TGT CCT CTC TCC and TCC AGA GCT CTC CTC ACC GAT G.

### 4.6. Hematoxylin and Eosin Staining and Immunohistochemistry

Tumor samples were dissected and fixed in 4% paraformaldehyde solution (Sangon Biotech, Shanghai, China). Tissues were embedded in paraffin (Sangon Biotech). Sections of tumors were cut at 3–4 μm and prepared for hematoxylin and eosin staining and immunohistochemistry by standard procedures. Deparaffinization and rehydration were conducted before antigen retrieval was performed using citrate antigen retrieval solution (pH = 6.0) for 20 min at 100 °C, with incubation using a rabbit polyclonal anti-cleaved caspase 3 antibody (Cell Signaling Technology, Danvers, MA, USA), an anti-Ki67 antibody (Abcam, Cambridge, UK), and anti-c-Myc (Abcam, Cambridge, UK) overnight at 4 °C in a humidified chamber, and using a second antibody (Zsbio, Beijing, China) for 30 min at room temperature. Reactions were visualized with DAB as substrate (Zsbio, Beijing, China). Finally, the sections were counterstained with Harris hematoxylin (Sangon Biotech) and normal IgG (Sigma, Saint louis, USA) as a negative control. Image acquisition was performed with a DM4B microscope (Leica, Germany).

### 4.7. Immunoblot analysis

Tumor or liver tissues and cells were lysed by 1% SDS lysis buffer and boiled at 95 °C for 10 min, then centrifuged at 12,000 rpm for 10 min at room temperature. The protein concentration was determined by a BCA protein assay reagent kit (Thermo Scientific, Waltham, MA, USA). The protein (35 μg) was separated by SDS-PAGE gels and transferred onto PVDF membranes (Millipore, Billerica, MA, USA). The membranes were blocked for 2h by 5% fat free milk in TBST at room temperature, followed by incubation overnight at 4 °C with the primary antibodies for anti-Glut1 (Abcam, Cambridge, UK), anti-Bcl2 antibody (Abcam), anti-Pparg antibody (Proteintech, Wuhan, Hubei, China), anti-Acaa1 (Proteintech), anti-Acadm (Proteintech,), anti-CD36 (Bioworld, Nanjing, China), anti-Hadh (Proteintech), anti-RXRA (Proteintech), anti-ACOX1 (Proteintech), and anti-β-actin (Sigma), respectively. The membranes were washed with TBST and incubated for 1 h with the corresponding HRP-conjugated second antibodies. Bands were visualized with ECL Reagents (Merck, Billerica, MA, USA).

### 4.8. Quantification of Glucose, Triglyceride, and NEFA in Serum

Serum was collected from mice treated with 0.9% NaCl and 50 mg/kg DEX (*n* = 3 mice, per group). The levels of glucose, triglycerides, and NEFAs in serum were determined by LabAssay^TM^ Glucose kits, LabAssay^TM^ Triglyceride kits, and LabAssay^TM^ NEFA kits, respectively (Wako, Japan), according to the manufacturer’s instructions.

### 4.9. Statistical Analysis

All experiments were repeated independently three times. Graphs and statistical analysis were generated using Prism 7 (GraphPad Software, lnc., La Jolla, CA, USA). The results were presented as mean ± SEM and statistical significance was analyzed by unpaired Student’s *t* test. Significance was indicated by ns, *p* > 0.05; _*_, *p* < 0.05; _**_, *p* < 0.01; _***_, *p* < 0.001.

## 5. Conclusions

Our data show that treatment with high-dose DEX significantly inhibits tumor progression in LLC cell line induced immunocompetent C57BL/6 mice, and the relevant mechanism may be associated with integrated M1-like macrophage polarization and the regulation of microenvironmental internal metabolic pathways relevant to glycolysis, TG synthesis, FFA import, and FAO, eventually leading to the inhibition of tumor progression.

## Abbreviations

GCs	Glucocorticoids
DEX	Dexamethasone
LLC	Lewis lung carcinoma
TME	The tumor microenvironment
MDSCs	Myeloid-derived suppressor cells
Tregs	Regulatory T cells
TAMs	Tumor-associated macrophages
Pparg	Peroxisome proliferator activated receptor Gamma
FFA	Free fatty acids
FAO	Fatty acid oxidation
VEGF	Vascular endothelial growth factor
Glut1	Glucose transporter 1
Hk2	Hexokinase 2
Pgk1	Phosphoglycerate kinase 1
Idh3a	Isocitrate dehydrogenase subunit alpha
Gpam	Glycerol-3-phosphate acyltransferase
Agpat2	1-acylglycerol-3-phosphate O-acyltransferase 2
Dgat1	Diacylglycerol O-acyltransferase 1
Cpt1a	Carnitine palmitoyltransferase 1A
Acadm	Acyl-CoA dehydrogenase medium chain
Pnpla2	Patatin-like phospholipase domain containing 2
Acaa1	Acetyl-CoA acyltransferase 1
Hadh	Hydroxyacyl-CoA dehydrogenase
Fabp1	Fatty acid binding protein 1
Slc27a4	Solute carrier family 27 member 4
NEFA	Non-esterified fatty acid
TCA	Tricarboxylic acid cycle
Pparα	Proliferator-activated receptor-alpha
RXRA	Retinoid X receptor alpha
ACOX1	Peroxisomal acyl-coenzyme A oxidase 1
PCK2	Phosphoenolpyruvate carboxykinase2
qPCR	Real-time quantitative PCR
ATP	Adenosine triphosphate
LABP	L-Arabinose binding protein
